# An on-chip deformability checker demonstrates that the severity of iron deficiency is associated with increased deformability of red blood cells

**DOI:** 10.1038/s41598-025-05483-2

**Published:** 2025-06-06

**Authors:** Kenji Kajitani, Tomohito Ohtani, Rie Higuchi, Misato Chimura, Fusako Sera, Chia-Hung Dylan Tsai, Yasutaka Ueda, Jun-ichi Nishimura, Yasushi Sakata

**Affiliations:** 1 Department of Cardiovascular Medicine, Graduate School of Medicine, The University of Osaka, 2-2 Yamadaoka, Suita, 565-0871 Japan; 2 Department of Mechanical Engineering, National Yang Ming Chiao Tung University, Hsinchu, Taiwan; 3 Department of Hematology and Oncology, Graduate School of Medicine, The University of Osaka, Suita, Japan

**Keywords:** Deformability, Iron deficiency, Red blood cell, Morphology, Ferritin, Microfluidics, Blood flow

## Abstract

The deformability of red blood cells (RBCs) is essential for peripheral circulation and RBC survival and is reportedly altered in several diseases. However, its detail in iron-deficiency (ID) anemia remains poorly understood. We investigated the association between ID and RBC deformability in 120 participants classified into four groups according to their ferritin and hemoglobin levels: non-ID/non-anemia (*n* = 61), ID/non-anemia (*n* = 32), ID/anemia (*n* = 15), and non-ID/anemia (*n* = 12). In the analysis using the established on-chip deformability checker, the normalized transit velocity of RBCs through the peripheral-vessel constriction models was significantly higher in the ID/anemia group than in the other groups. The RBC deformability index (RDI), derived from the normalized transit velocity and degree of RBC deformation, was highest in the ID/anemia group, followed by the ID/non-anemia group. The RDI was negatively correlated with log_10_ ferritin levels (*r* = -0.66, *p* < 0.01), even after adjusting for hemoglobin levels. Lower log_10_ ferritin levels correlated with thinner, more oval-shaped RBCs and lower internal viscosity. These RBC characteristics were significantly associated with a higher RDI. These results suggest that, among several known determinants of RBC deformability, RBC morphology and internal viscosity are altered in ID, resulting in higher deformability.

**Supplementary Information:**

The online version contains supplementary material available at 10.1038/s41598-025-05483-2.

## Introduction

Red blood cells (RBCs) are flat, biconcave, disk-shaped, anucleate cells, approximately 8 μm in diameter and 2 μm thick, and play a pivotal role in transporting oxygen from the lungs to peripheral tissues^1^. The highly deformable properties of RBCs allow them to pass through narrow passages smaller than their diameter, such as capillaries and the interendothelial slits of the spleen, with reduced physical stress in the microcirculation and without becoming trapped or damaged^2^, which is an important factor for RBC survival. RBC deformability is influenced by internal factors such as membrane stiffness, morphology, and internal viscosity^3^ and external factors such as osmotic pressure^4^ and various mediators ^5,6^. These factors are affected by various pathological conditions, including iron-deficiency (ID) anemia. RBCs in ID anemia usually have a low mean corpuscular volume (MCV) and low mean corpuscular hemoglobin concentration (MCHC). They contain thin and flat RBCs called leptocytes or oval RBCs called elliptocytes. These characteristics of RBCs may influence their deformability; however, their relevance remains poorly understood. In addition, a recent study revealed that the RBC lifespan of patients with ID anemia was longer than that of healthy individuals^7,8^. Considering that RBC deformability can affect the lifespan of RBCs, it is anticipated that RBCs from patients with ID anemia will have increased deformability. However, RBC deformability in ID anemia remains poorly understood.

The transit velocity of RBCs through a constricted channel is conventionally used as an index of cellular deformability. The resistance RBCs experience in a constriction can be explained by Thin-Film Lubrication Theory ^9–11^. A thin layer of fluid exists between the wall and the RBC surface, preventing direct contact. The resistance force arises from the shear stress within this fluid layer. A low deformability forms a thinner fluid layer, increasing resistance from the constriction wall and leading to faster velocity in the constriction, and vice versa. Based on this principle, we established a microfluidic system with constrictions of different widths to simulate capillary vessels and evaluate RBC deformability^12^. The transit velocity was determined by fluid viscosity and composition, flow rate, cell size, constriction size, and cell deformability. In this study, the fluid viscosity and composition were held constant. The transit velocity was normalized by the flow rate, and the degree of cell deformation was calculated based on the cell and constriction size. In this context, the normalized transit velocity was determined by the degree of cell deformation and deformability. When the degree of deformation is specified at an arbitrary value, the normalized transit velocity depends primarily on RBC deformability. In this study, the normalized transit velocity corresponding to 50% of the maximum deformation was employed for the quantitative assessment of RBC deformability.

We aimed to evaluate RBC deformability in ID anemia and investigate the association of RBC deformability with ferritin levels, which are used to diagnose and assess the severity of ID, and RBC parameters, including morphology and internal viscosity, which are important determinants of RBC deformability.

## Methods

### Study participants

We prospectively screened individuals who were admitted to Osaka University Hospital or visited the Osaka University Health Center and underwent blood collection for medical purposes. We included participants whose blood samples were available for the experiment within 6 h of collection and who consented to participate in this study. Exclusion criteria included chronic kidney disease with estimated glomerular filtration rate (eGFR) < 30 mL/min/1.73 m^2^, heart failure with BNP > 100 pg/mL or NT-pro BNP > 400 pg/mL, any signs of infection, history of malignancy, chronic inflammatory disease, and hereditary blood disorders. Finally, this study included a total of 120 participants. All patients underwent laboratory tests, including complete blood count, iron profile, liver function, renal function, and diabetic status. Patient characteristics, clinical histories, and medication information were collected. ID was defined as a low ferritin level (< 30 ng/mL)^13^ and anemia was defined as a low hemoglobin level (< 13 g/dL for men and < 12 g/dL for women)^14^. Participants were classified into four groups based on the presence or absence of ID and anemia: control (non-ID/non-anemia), ID/non-anemia, ID/anemia, and non-ID/anemia. All participants provided written informed consent to participate in this study, which was approved by the ethics committee of Osaka University Hospital (IRB number 16239-8). This study conformed to the ethical guidelines outlined in the Declaration of Helsinki.

### Sample preparation

Venous blood was collected via venipuncture and placed into heparin vacutainer tubes. An aliquot of 5 µL was washed three times with phosphate-buffered saline (PBS) (1 mL) and 1% w/v bovine serum albumin (BSA) to remove plasma (500 g for 5 min). Pelleted RBCs were re-suspended in PBS (100 µL) with 1% BSA. BSA was used to stabilize the RBC morphology^15^ and prevent RBC adhesion to the microfluidic device^16^. Additionally, beads of 2 μm (L0280, Sigma-Aldrich) were added as an internal control to estimate the flow rate through the microfluidic passage.

### Experimental system

**Fig. 1:**
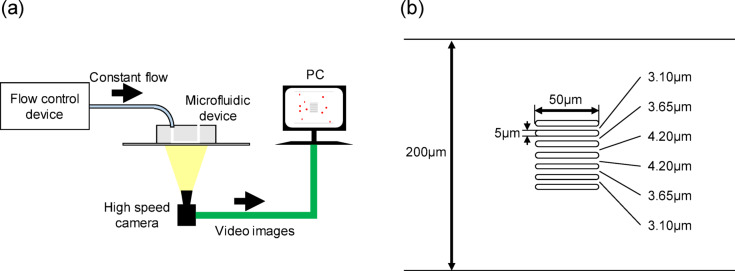
Schematic representation of the microfluidic system for measuring RBC deformability. (**a**) The microfluidic device is connected to a pressure control system. The pressure control system provides a constant pressure source for stable fluid flow in the microfluidic chip. A high-speed vision camera is installed on the microscope to record images of RBCs and beads. (**b**) Dimensions of microfluidic passage and constrictions. The microfluidic passage is 200 μm wide and 3.5 μm deep. Six obstacles with 50 μm in length and 5 μm in width are located parallel in the center of the passage to make constrictions between the obstacles. The constriction widths are 3.10, 3.65, 4.20, 4.20, 3.65, and 3.10 μm from top to bottom, respectively.

The experimental system comprised three main parts: a microfluidic chip, a pressure control system (FlowEZ^®^, FLUIGENT), and a high-speed vision system (IDP, Photron Co.) (Fig. 1a). A microfluidic chip made of polydimethylsiloxane was fabricated using photolithography techniques, as detailed in our previous study^12^. The accurate geometry of the device was measured using a laser microscope (OLS-4100, Olympus) (Fig. 1b). An aliquot of the blood sample containing the beads was loaded into the microfluidic device inlet and pushed into the passage with positive pressure. The process of RBCs flowing through the passage, with some entering the constrictions, was captured using a high-speed vision camera (Supplemental video 1). A sequence of images was imported into a video editing software (AviUtl) to measure the size and the velocity of RBCs and beads. In this system, the flow rate was controlled to prevent RBC deformation.

### Variables representing the morphology and internal viscosity of RBCs

**Fig. 2:**
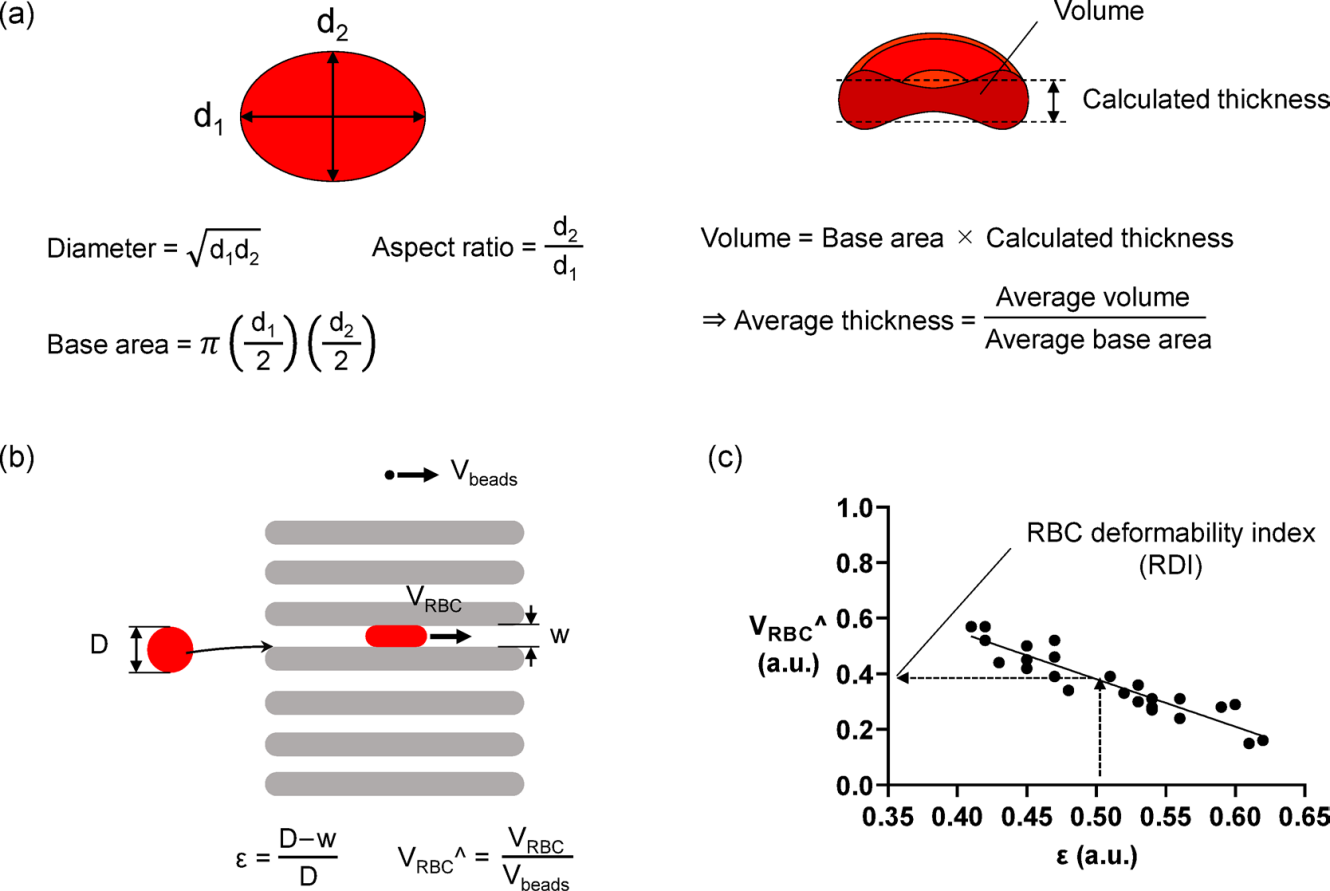
Evaluation for RBC morphology and deformability. (**a**) RBC morphological parameters. The RBC diameter was calculated from the lengths of the two principal axes of the RBC as $\:\sqrt{{d}_{1}{d}_{2}}$, where d_1_ and d_2_ are the long and short axes, respectively. The aspect ratio was calculated as d_2_/d_1_. (**b**) Parameters used to evaluate RBC deformability. The black dots and red circles represent beads and RBC, respectively. RBC deformation, ɛ, was calculated as ɛ = (D-w)/D, where *w* is the width of the constriction the RBC passed through. The normalized transit velocity of the RBC in constriction, V_RBC_^, was calculated as V_RBC_^ = V_RBC_/V_beads_, where V_RBC_ is the transit velocity of the RBC through the constriction, and V_beads_ is the bead velocity outside the constrictions. (**c**) The RBC deformability index (RDI). The relationship of ɛ and V_RBC_^ was plotted on the graph, with ɛ on the X-axis and V_RBC_^ on the Y-axis. A straight line indicates an approximately linear trend. RDI was defined as the value of V_RBC_^ at ɛ = 0.5.

MCV and MCHC obtained from blood tests were used as indices for RBC morphology and internal viscosity, respectively. To evaluate RBC morphology in detail, we calculated diameter, aspect ratio, and thickness as shown in the following steps. For each RBC entering the constriction, the lengths of the two principal axes of the RBC, long axis (d_1_) and short axis (d_2_), were measured before entering the constrictions. RBC diameter was defined as the geometric mean of the two axes. The aspect ratio of the RBC was calculated as d_2_/d_1_, which is the ratio between the length of the major and minor axes. The average thickness of the RBCs was calculated based on the principle that volume is expressed as the product of the base area and thickness, as described elsewhere^17^. The base area of test RBC was calculated as π(d_1_/2)(d_2_/2), and the average base area was obtained from all test RBCs. The average thickness was calculated as (average volume)/(average base area) (Fig. 2a).

### Evaluation of RBC deformability

The deformation and transit velocity of RBCs in the constriction were evaluated, as shown in Fig. 2b. RBC deformation was normalized as the ratio of the amount of deformation to its original size, defining it as the deformation (ɛ). The velocities of RBC through the constrictions were normalized by the velocity of the beads passing outside the constrictions, defined as the normalized transit velocity of the RBC (V_RBC_^). RBC deformability was evaluated, as shown in Fig. 2c. In this study, the RBC deformability index (RDI) was defined as the value of V_RBC_^ when ɛ is 0.5 in the regression line between ɛ and V_RBC_^. The intra- and inter-rater agreements of the RDI were analyzed by calculating the intraclass correlation coefficients in 10 randomly selected participants. The intra-rater agreement was calculated as the agreement between the first and second measurements by a single observer (KK), while the inter-rater agreement was calculated as the agreement between the first measurements by two observers (KK and HR). The intraclass correlation coefficients for intra- and inter-observer agreement of the RDI were 0.982 (95% CI: 0.935–0.996, *p* < 0.001) and 0.966 (95% CI: 0.876–0.991, *p* < 0.001), respectively.

### Statistical analysis

Categorical variables were presented as numbers (percentages), and continuous variables were expressed as the mean ± standard deviation (SD) if symmetrically distributed, or as the median (interquartile range [IQR]) if asymmetrically distributed. To investigate the associations between the groups and relevant variables, multiple comparisons were performed using one-way analysis of variance or the Kruskal–Wallis test for continuous variables, as appropriate. Fisher’s exact test was used for categorical variables. For all pairwise comparisons, Tukey–Kramer and Steel–Dwass tests were used for continuous variables, and Fisher’s exact test with Bonferroni correction was used for categorical variables. Variables that were significantly different among the groups were analyzed for their correlation with the RDI in univariate and multivariate analyses. Ferritin and hemoglobin levels were selected as representative iron parameters and RBC indicators, respectively. The ferritin value was log-transformed (log_10_) for linearization. The relationships between the RDI and the parametric and nonparametric variables were assessed using Pearson and Spearman rank correlation coefficients (r). All variables used in the univariate analysis were included in the multivariate analysis. A p-value < 0.05 was considered statistically significant. All statistical analyses were performed using the JMP (version 17.1) software package.

## Results

### Clinical characteristics

Table 1 shows the clinical characteristics of the four groups. Compared to the control group, the ID/non-anemia and ID/anemia groups had more women and compatible laboratory data related to ID or anemia, including hemoglobin and ferritin levels. Notably, the ID/anemia group had lower ferritin levels than the ID/non-ID anemia group. The levels of aspartate aminotransferase, eGFR, total cholesterol, and C-reactive protein differed slightly among the four groups but were within normal limits.

**Table 1:** Clinical characteristics of participants grouped by ID and anemia status.

	Control	ID/non–anemia	ID/anemia	Non–ID/anemia	p-value
	(*n* = 61)	(*n* = 32)	(*n* = 15)	(*n* = 12)	
General information					
Age, years	51 (41–63)	45 (40–51)	46 (40–51)	73 (48–81)†	< 0.01
Women, n (%)	29 (48%)	28 (87%)*	13 (87%)*	9 (75%)	< 0.001
BMI, kg/m^2^	21.9 (20.2–24.2)	21.7 (19.6–23.7)	21.2 (19.9–22.0)	20.1 (18.4–22.3)	0.35
Systolic BP, mmHg	124 ± 14	119 ± 16	120 ± 13	123 ± 20	0.49
Laboratory data					
RBC count, 10^6^/µL	4.64 ± 0.45	4.46 ± 0.36	4.25 ± 0.30*	3.67 ± 0.75*†‡	< 0.001
Hemoglobin, g/dL	14.3 ± 1.3	13.1 ± 0.8*	10.7 ± 1.1*†	10.7 ± 1.3*†	< 0.001
Hematocrit, %	43.9 ± 3.5	40.7 ± 2.6*	34.9 ± 3.0*†	33.6 ± 4.6*†	< 0.001
Iron, µg/dL	104 (87–121)	92 (63–123)	28 (16–44)*†	81 (48–111)‡	< 0.001
TIBC, µg/dL	326 ± 45	375 ± 67*	414 ± 57*	261 ± 56*†‡	< 0.001
UIBC, µg/dL	217 (178–254)	275 (216–332)*	405 (356–428)*†	179 (123–201)†‡	< 0.001
Ferritin, ng/mL	97 (52–156)	10 (7–16)*	4 (2–6)*†	86 (51–143)†‡	< 0.001
AST, IU/L	22 (19–26)	19 (17–22)	17 (14–20)*	19 (17–28)	< 0.01
eGFR, mL/min/1.73 m^2^	78 ± 14	84 ± 18	81 ± 15	64 ± 22*†‡	< 0.01
Total cholesterol, mg/dL	218 (189–250)	190 (173–207)*	189 (158–197)*	192 (146–197)*	< 0.001
Glucose, mg/dL	93 (85–100)	87 (80–93)	87 (84–99)	89 (86–181)	0.09
Hemoglobin A1c, %	5.3 (5.1–5.5)	5.2 (5.1–5.7)	5.5 (5.2–6.0)	5.5 (4.8–8.8)	0.24
CRP, mg/dL	0.04 (0.02–0.09)	0.03 (0.01–0.06)	0.02 (0.01–0.05)	0.07 (0.04–0.61)†‡	0.02

Categorical variables are expressed as numbers (percentages). Continuous variables are expressed as mean ± standard deviation if symmetrically distributed, or as median (interquartile range) if asymmetrically distributed. *p < 0.05 vs. control. †*p* < 0.05 vs. ID/non-anemia. ‡*p* < 0.05 vs. ID/anemia. AST, aspartate aminotransferase; BMI, body mass index; BP, blood pressure; CRP, C-reactive protein; eGFR, estimated glomerular filtration rate; ID, iron deficiency; RBC, red blood cell; TIBC, total iron-binding capacity; UIBC, unsaturated iron-binding capacity.

### Impact of ID on RBC deformability

**Fig. 3:**
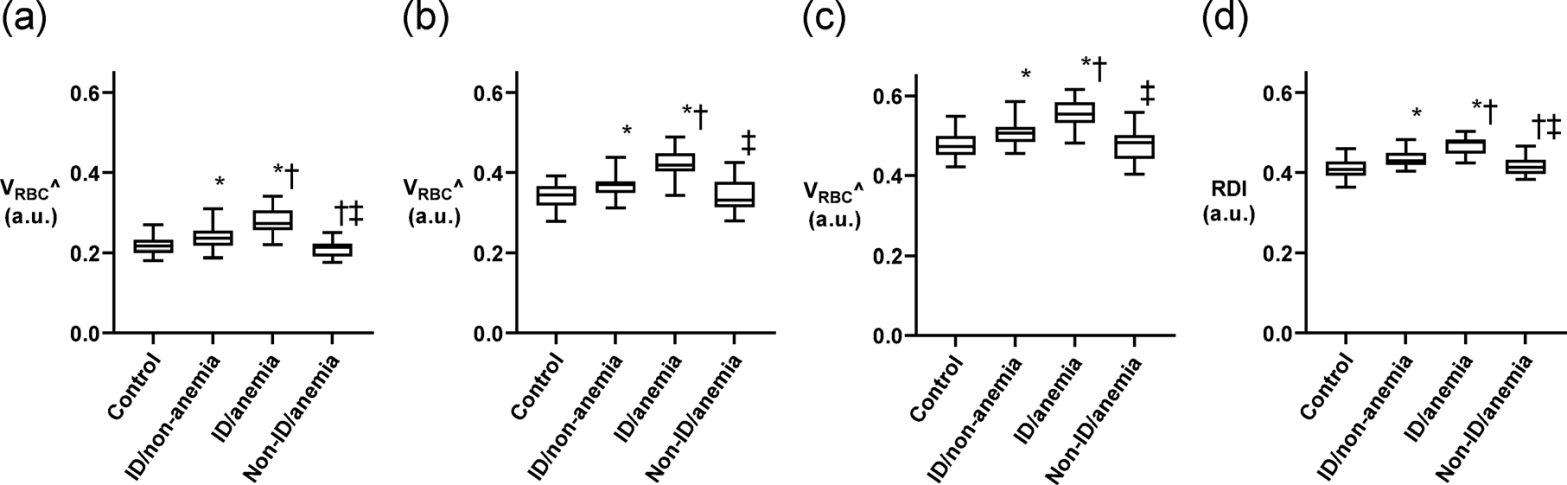
Comparison of V_RBC_^ and RDI according to the presence or absence of ID and anemia. The values of V_RBC_^ in the constrictions with widths of (**a**) 3.10 μm, (**b**) 3.65 μm, and (**c**) 4.20 μm, and (**d**) RDI are shown in the box plots. The line across each box represents the median value. The top and bottom of each box represent the first and third quartiles, respectively. Whiskers exhibited the highest and lowest values. *p < 0.05 vs. control. †*p* < 0.05 vs. ID/non-anemia. ‡*p* < 0.05 vs. ID/anemia; V_RBC_^, normalized transit velocity of RBC in constriction; RDI, RBC-deformability index.

**Fig. 4:**
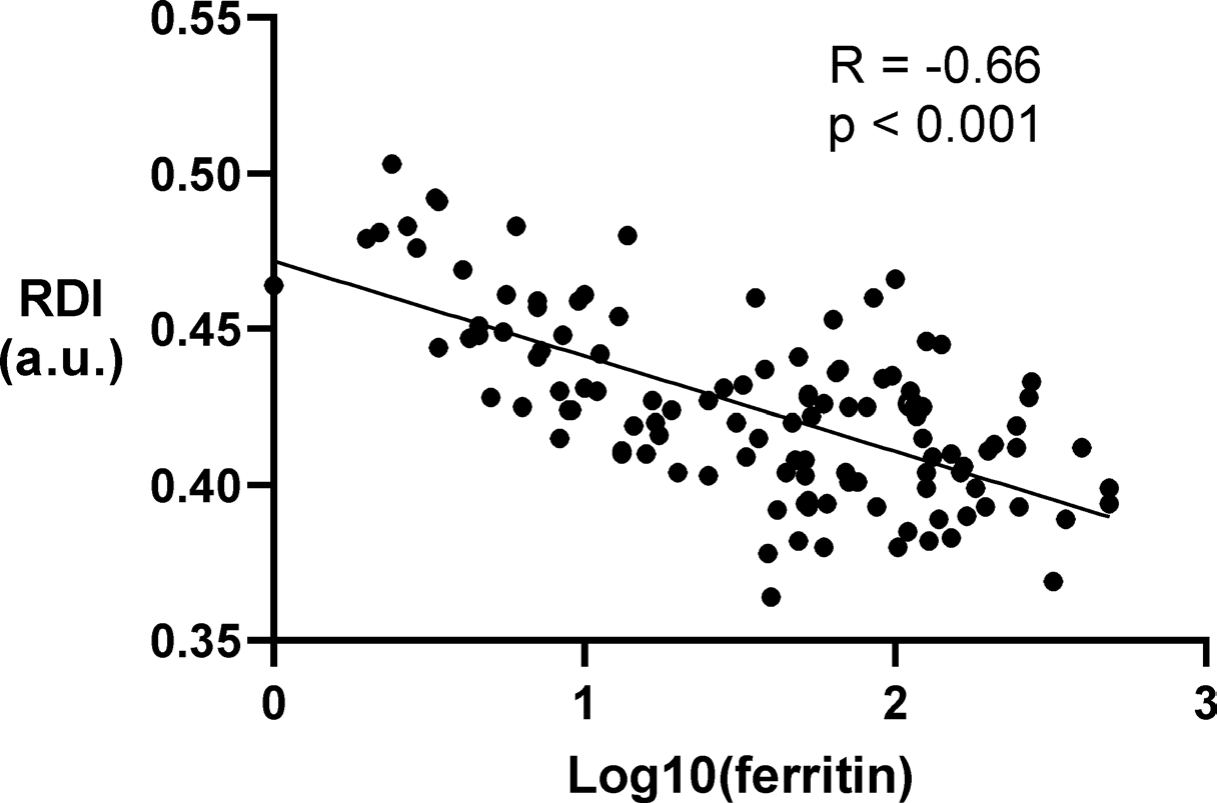
Correlation of ferritin levels to RDI. Scatter plots show the correlation between logarithmically transformed ferritin levels and RDI. Regression analysis indicated a significant interaction (*p* < 0.001) and a negative correlation (*r* = −0.66). RDI, RBC deformability index.

The V_RBC_^ for each constriction width is shown in Fig. 3a and b, and 3c. The differences in V_RBC_^ among the four groups were consistent across the three constriction widths. Patients in the ID/anemia group had the highest V_RBC_^ for each constriction width, whereas patients in the non-ID/anemia group had V_RBC_^ similar to that of the control group, indicating that ID, rather than anemia, affects V_RBC_^. The differences in RDI among the four groups were consistent with those in V_RBC_^ (Fig. 3d), and patients in the ID/anemia group had the highest RDI. Patients in the ID/non-anemia group had a higher RDI than those in the control and non-ID/anemia groups, indicating that ID increases RDI. Table 2 shows the association between RDI and clinical characteristics. In univariate regression analysis, sex, hemoglobin level, log_10_ ferritin level, aspartate aminotransferase level, and total cholesterol level were associated with RDI. Multivariate regression analysis demonstrated that only log_10_ ferritin was an independent determinant of RDI (standard β = −0.65, *p* < 0.001). The correlation between RDI and log_10_ ferritin levels was strong (*p* < 0.001, *r* = −0.66, Fig. 4), indicating that the more severe the ID, the higher the RDI.

**Table 2:** Associations between the clinical characteristics and RDI.

	Univariate		Multivariable	
	Correlation Coefficient	p-value	Standard β	p-value
General information				
Age, years		0.73		
Females, n (%)		< 0.01		0.11
Laboratory data				
Hemoglobin, g/dL	−0.42	< 0.001	−0.19	0.04
Log_10_ Ferritin	−0.66	< 0.001	−0.65	< 0.001
AST, IU/L	−0.26	< 0.01		0.47
eGFR, mL/min/1.73 m^2^		0.17		
Total cholesterol, mg/dL	−0.26	< 0.01		0.90
CRP, mg/dL		0.43		

AST, aspartate aminotransferase; CRP, C-reactive protein; eGFR, estimated glomerular filtration rate; RDI, RBC-deformability index.

### RBC characteristics bridging ID and RBC deformability

**Fig. 5:**
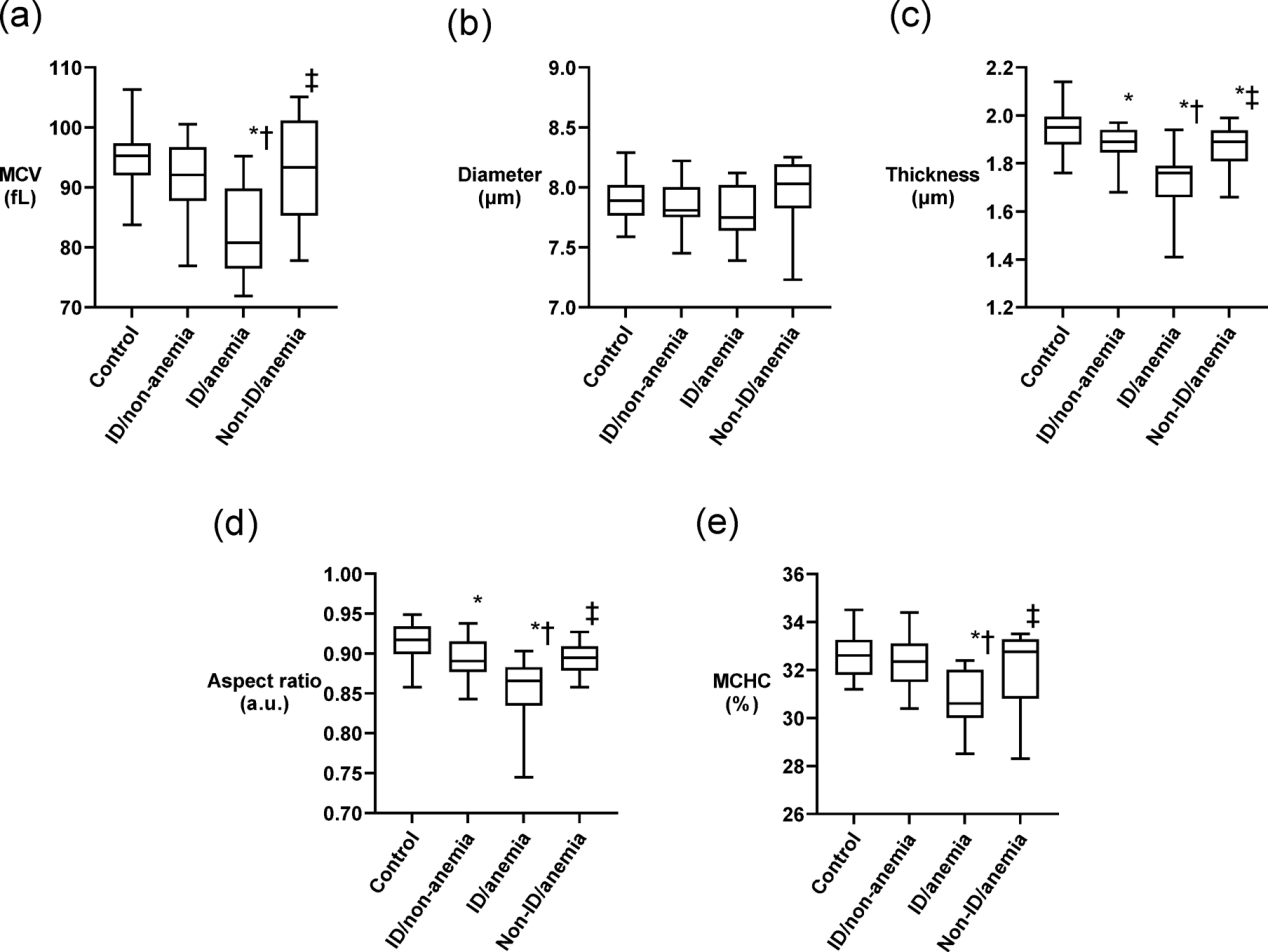
Comparison of RBC parameters according to the presence or absence of ID and anemia. (**a**) MCV, (**b**) diameter, (**c**) thickness, (**d**) aspect ratio, and (**e**) MCHC of the four groups are shown as box plots. The line across each box represents the median value. The top and bottom of each box represent the first and third quartiles, respectively. Whiskers exhibited the highest and lowest values, respectively. *p < 0.05 vs. control. †*p* < 0.05 vs. ID/non-anemia. ‡*p* < 0.05 vs. ID/anemia; MCV, mean corpuscular volume; MCHC, mean corpuscular hemoglobin concentration.

The differences in RBC parameters among the four groups are shown in Fig. 5. The ID/anemia group had lower MCV, thickness, aspect ratio, and MCHC than the control group, and lower MCV, thickness, and MCHC than the non-ID/anemia group. Although the non-ID/anemia group had a slightly lower thickness and aspect ratio than the control group, all parameters in the group were more similar to those in the control group than those in the ID/anemia group. The ID/non-anemia group had a lower thickness and aspect ratio than the control group, indicating morphological changes in patients with ID, even in the absence of anemia. RBC diameter did not differ among the four groups.

Log_10_ ferritin levels positively correlated with the following RBC parameters: MCV (*p* < 0.001, *r* = 0.45), diameter (*p* = 0.02, *r* = 0.22), thickness (*p* < 0.001, *r* = 0.43), aspect ratio (*p* < 0.001, *r* = 0.51), and MCHC (*p* < 0.001, *r* = 0.36). MCV, thickness, aspect ratio, and MCHC were negatively correlated with RDI (*p* < 0.001, *r* = −0.45; *p* < 0.001, *r* = −0.62; *p* < 0.001, *r* = −0.63; *p* < 0.001, *r* = −0.40, respectively). In contrast, when analyzed only in patients without ID (control and non-ID/anemia groups, *n* = 47), log_10_ ferritin levels were not correlated with these RBC parameters. However, their MCV, thickness, and aspect ratio were negatively correlated with RDI (*p* < 0.03, *r* = −0.25; *p* < 0.001, *r* = −0.43; *p* < 0.001, *r* = −0.45, respectively).

## Discussion

This study is the first to demonstrate an association between ID severity and RBC deformability using an on-chip analysis system with capillary vessel models. This study had two major findings. First, decreased ferritin levels are associated with greater RBC deformability, suggesting that RBCs in ID anemia are more likely to pass through narrow capillaries. Notably, this change in deformability was observed in patients with ID who did not present with anemia. Second, decreased ferritin levels were associated with thinner and more oval-shaped RBCs and lower internal viscosity. These characteristics of the RBCs are associated with their increased deformability. These results suggest that ID affects RBC deformability through changes in the RBC characteristics.

Several studies using ektacytometry reported that RBCs in ID anemia have low deformability^18–20^. In ektacytometry, RBC deformability is evaluated by measuring their elongation in the shear flow of a highly viscous solution. In contrast, the microfluidic system used in this study simulates RBC behavior in peripheral vessels, where the RBCs are compressively deformed. The differences in the underlying principles of the deformability measurements may explain these contrasting results. Reinhart et al. reported that RBCs in ID anemia showed high deformability using the filtration method^21^. In this method, filters with pores smaller than the RBC diameter are used, and RBCs are compressively deformed when passing through the pores. This principle is similar to that of our measurement system, and the results are consistent with those of this study.

Compared with the filtration method, the advantage of the microfluidic system used in this study is that it allows direct measurement of RBC transit velocity and captures a wider range of RBC deformations using three different constriction widths. Additionally, because the size of RBCs can be observed under a microscope, it is possible to illustrate the relationship between the deformation and normalized transit velocity, allowing a quantitative evaluation of RBC deformability. RBC morphology is a determinant of RBC deformability^5^. In this study, we found significant associations between MCV, thickness, aspect ratio, and deformability. These morphological changes are consistent with the common observations of thin (leptocytes) and elongated RBCs (elliptocytes) in peripheral blood smears of patients with ID anemia^22^. Furthermore, we found an association between these morphological changes and deformability in patients with a pre-anemic state of ID, which has not been sufficiently studied. Meanwhile, in patients without ID, RBC morphology including MCV, thickness, and aspect ratio were associated with deformability, whereas ferritin levels were not. In our preliminary study with patients with thalassemia and hereditary spherocytosis, RBC deformability differed by different RBC thickness even at similarly low levels of MCV (data not shown). These findings suggest that detailed morphological changes beyond MCV, including thickness and aspect ratio, affect RBC deformability, and which morphological change is a key determinant may vary in different diseases.

When RBCs pass through the constrictions, they are forced to bend and fold to fit the constriction width. Bending rigidity, which indicates the resistance to deformation, decreases as the material thickness decreases; thus, thinner materials have lower bending rigidity and are more easily deformed^23^. Although the orientation of the long axis of RBCs before they enter the constrictions varies, there is an 80% or greater likelihood that their long axis will align with the flow direction upon contact with the constriction entrance^12^. This phenomenon is commonly observed in the ID/anemia group, as their RBCs include elliptocytes (supplemental video 2). In this situation, the short axis is compressed by the stenotic wall, and the oval erythrocytes are considered more physically deformable. The internal viscosity of RBCs is also considered to be a determinant of RBC deformability^5^. In this study, a lower MCHC was correlated with higher deformability. Since the internal fluid of RBCs is rich in hemoglobin, a decrease in the MCHC leads to a decrease in the internal viscosity^24^. The study’s results on ID were consistent with the idea that RBC morphology, thin and elongated shapes, and low internal viscosity are associated with high deformability.

Interendothelial slits in the spleen act as primary blood filters. RBCs with reduced deformability cannot pass through these slits and are subsequently destroyed by macrophages^25,26^. This process constantly controls the quality of the circulating RBCs. Recent studies have shown that the RBCs in patients with ID anemia have longer lifespans than those in healthy individuals^7,8^. Although the mechanism remains unclear, our findings suggest that in ID anemia, the increased deformability of RBCs may reduce their likelihood of being trapped in interendothelial slits, thereby extending their lifespan. This potential mechanism may compensate for the reduced hematopoietic ability in patients with ID.

### Limitation

This study had a few limitations. First, RBCs exist in vivo at approximately 37 ℃; however, the experiments were conducted at 26 ℃. In addition, the experimental solution contained 1% w/w of protein (BSA), whereas plasma contained approximately 8% w/w of protein, indicating that its fluid viscosity, a determinant of transit velocity, was lower than that of plasma. Although our preliminary data suggested no distinct change in RDI at different temperatures (data not shown), the experimental system did not fully replicate the in vivo environment. Second, the membrane rigidity of RBCs affects their deformability and was not evaluated. Third, further study is warranted because the association between deformability measured by our system and other methods, such as ektacytometry, has not been evaluated. Finally, this was a cross-sectional study, and serial data on RBC deformability related to changes in iron status were not evaluated.

## Conclusions

ID correlates with the high deformability of RBCs. This high deformability can be explained by the morphological characteristics and low internal viscosity of RBCs, which are associated with ID. This finding implies that the increased deformability of RBCs in ID may reduce their likelihood of being trapped in interendothelial slits in the spleen, potentially extending RBC lifespan.

## Electronic supplementary material

Below is the link to the electronic supplementary material.

Supplementary Video 1

Supplementary Video 2

## Data Availability

The datasets used or analyzed during the current study are available from the corresponding author upon reasonable request.
